# Mechanism of Sishen-Pill-Regulated Special Memory T and mTfh Cell via Involving JAK/STAT5 Pathway in Colitis Mice

**DOI:** 10.1155/2022/6446674

**Published:** 2022-03-28

**Authors:** Mengxue Wang, Xiaoying Huang, Zengping Kang, Jiaqi Huang, Siyi Wei, Haimei Zhao, Youbao Zhong, Duanyong Liu

**Affiliations:** ^1^Graduate School, Jiangxi University of Chinese Medicine, Nanchang 330004, Jiangxi, China; ^2^Key Laboratory of Modern Preparation of TCM, Ministry of Education, Jiangxi University of Chinese Medicine, Nanchang 330004, Jiangxi, China; ^3^College of Traditional Chinese Medicine, Jiangxi University of Chinese Medicine, Nanchang 330004, Jiangxi, China; ^4^Experimental Animal Science and Technology Center, Jiangxi University of Chinese Medicine, Nanchang 330004, Jiangxi, China; ^5^Formula-Pattern Research Center of Jiangxi University of Chinese Medicine, Nanchang 330004, Jiangxi, China

## Abstract

It is known that memory T cells (mT cell) and memory T follicular cells (mTfh) play vital roles in the IBD pathogenesis. Sishen Pill (SSP) is a classic prescription used to treat chronic ulcerative colitis (UC). However, it is still unclear whether SSP can regulate immune homeostasis induced by mT cell and mTfh to treat IBD. In this study, we measured mT cell and mTfh level to explore the conceivable mechanism of SSP-treated IBD. The mice colitis were induced by dextran sulfate sodium (DSS) and were treated by SSP for 7 days. The therapeutic effect of SSP was evaluated by macroscopic and microscopic observation; the mT cell, mTfh, and their subsets were analyzed by flow cytometry. Activation of the JAK/STAT signaling pathway was analyzed by using a Western blot. In the present study, SSP significantly reversed weight loss and colonic injury (colon weight increase and colonic length shortening) caused by 3% DSS in physiological saline solution. Flow cytometry showed that the percentages of CD4^+^ and CD8^+^ expressions on central memory T cells were enhanced after SSP treatment, while the CD4^+^ T cm, CD4^+^ mTfh (memory T follicular helper) cells and their subpopulations were also significantly increased. Moreover, SSP inhibited the expression of JAK/STAT signaling pathway proteins JAK1, PIAS3, STAT5, p-STAT5, BIM, BAX, caspase-3, and *β*-casein and promoted the expression of JAK3, PISA1, Bcl-2, and caveolin-1. In summary, SSP can regulate immune homeostasis induced by mT cell and mTfh in DSS-induced colitis, which is potentially correlated with JAK/STAT signaling pathway activation.

## 1. Introduction

Inflammatory bowel disease (IBD) is a group of chronic recurrent intestinal diseases involving the ileum, colon, and rectum, including ulcerative colitis (UC) and Crohn's disease (CD). The clinical symptoms include loss of body weight, abdominal pain, diarrhea, and bloody stools [1]. The etiology of IBD is complex and involves the coregulation of factors such as immunity, infection, genetics, and environment, among which immune dysfunction is known as the main factor [2]. In the initial stage, T cells are activated and differentiated into effector T cells. After binding with antigens and removing them, most of the effector T cells undergo apoptosis, while some become memory T cells. The memory T cells maintain and consolidate immune homeostasis to build immunologic balance. Memory T cells can quickly activate and defend against secondary antigen invasion. Once memory T cells function is imbalanced, secondary antigen stimulation may promote the differentiation of the memory T cells into the abnormal effective T cell and further induce autoimmune diseases, such as UC and CD [3]. Many researches had indicated that the JAK-STAT pathway is the main pathway transmitting signals from cytokines to influence the formation and maintenance of memory T cells, as STAT3 signal promoted cell long-term survival and the formation of self-renewed and protective memory CD8 T cells [4]. These studies hinted the JAK-STAT signaling pathway plays a significant role in the differentiation of memory T cells.

As a classic prescription of traditional Chinese medicine-treated UC, Sishen Pill (SSP) has anti-inflammatory and immunomodulatory effects and has been treating chronic enteritis in China for thousands of years [5]. For example, the clinical study of 204 patients with IBD found that the total effective rate of SSP was 75.98%, and the recurrence rate within 6 months was only 8.1%, while the recurrence rate with sulfasalazine was 23.3% [6]. In the previous study, we had performed the pharmacodynamic evaluation of the different doses (5, 2.5, and 1.25 g·kg^−1^) of SSP-treated colitis induced by TNBS in rats. The above results were reported in the *Journal of Ethnopharmacol* that the 2.5 g·kg^−1^ SSP effectively treated TNBS-induced colitis, which was the best dose to regulate the expression of IL-10 and IL-4 mRNA [7]. And these results were repeatedly proved in our previous other studies [8–10]. So, in the present study, we selected the 2.5 g·kg^−1^ SSP to explore the mechanism of SSP-treated colitis. While our research in 2020 has shown that SSP regulates the interaction between inflammatory DCs and gut microbiota and the differentiation and function of follicular T helper cell (Tfh) cells to treat IBD [10]. However, it is still unclear whether SSP can regulate immune homeostasis induced by special memory T cells (TSM) and memory T follicular cells (mTfh) to alleviate IBD. To explore the possible mechanism of SSP-treated experimental colitis, we observe the changed characters of TSM and mTfh cells by flow cytometry and the activation of the JAK/STAT5 signaling pathway by Western blot analysis.

## 2. Materials and Methods

### 2.1. Animals

Forty male BALB/c mice, weighing 22 ± 2 g, were purchased from Hunan Shrek Jingda Experimental Animal Co. Ltd. (Changsha, China; Permission No.: SCXK (Xiang) 2016-0002). All the animals were housed in an environment with a temperature of 22 ± 1°C, relative humidity of 50 ± 1%, and a light/dark cycle of 12/12 hr and were given food and water ad libitum. All animal studies (including the mice euthanasia procedure) were done in compliance with the regulations and guidelines of Jiangxi University of Chinese Medicine (JXUCM) institutional animal care and conducted according to the AAALAC and the IACUC guidelines (No.: JZ2018-139).

### 2.2. Drugs

DSS (No. 160110; molecular weight 36,000–50,000 kDa) was obtained from MP Biomedicals (California CA, USA). SSP (batch number 17080051, Tongrentang Natural Medicine Co. Ltd. (Beijing, China) was composed of *Euodia rutaecarpa* (Juss.) Benth., *Schisandra chinensis* (Turcz.) Baill., *Psoralea corylifolia* L., *Myristica fragrans* Houtt., *Ziziphus jujuba* Mill., and *Zingiber officinale* Rosc. (Pharmacopoeia of China (2015)), which were prepared into pills according to the dose ratio (100 g, 200 g, 400 g, 200 g, 200 g, and 200 g, ratio: 1:2:4:2:2:2, respectively). The main active ingredients of SSP were measured by high-performance liquid chromatography (HLPC) according to a previously described approach. The previous study of Zhang and his coworkers measured the main active ingredients of SSP by HPLC-ESI-MS/MS, which include isopsoralen (1,293.7 *μ*g/g), schizandrin (258.0 *μ*g/g), *γ*-schizandrin (131.5 *μ*g/g), psoralen (131.08 *μ*g/g), deoxyschizandrin (72.6 *μ*g/g), and so on [11]. Mesalazine (5-ASA) was obtained from Sunflower Pharmaceutical Group Jiamusi Luling Pharmaceutical Co. Ltd. (batch number 181003; Heilongjiang, China).

### 2.3. Experimental Design and Therapeutic Protocols

Mice were randomly divided into four groups (10 mice per group): Normal (Nor) group, the health mice without DSS administration; DSS group (DSS), the mice with DSS administration into colitis; Sishen pill treated (DSS + SSP) group, the colitis mice treated with SSP; and Mesalazine controlled (DSS + 5-ASA) group, the colitis mice treated with mesalazine. According to the previous literature [12], the mice in the Nor group had free access to drinking water, while other mice were treated with 3% (*w*/*v*) DSS solution prepared with the drinking water for 7 days to induce ulcerative colitis. On the 8^th^ day, according to the previous researches [8–10] and weight conversion human and mice on the basis of clinic dosages of SSP and 5-ASA, the mice in the DSS + SSP group were treated with 2.5 g/kg Sishen pill suspension, while the mice in the DSS + 5-ASA group were treated with 300 mg/kg mesalazine suspension [13]. The mice in the Nor group and DSS group were fed with the equivalent volume of physiological saline solution for 7 days. Their body hair, drinking water, and food intake, fecal traits, hematochezia, and body weight were monitored and analyzed once per day.

### 2.4. Macroscopical Observation

On day 15, all animals were sacrificed under sodium pentobarbital (50 mg/kg i.p.) anesthesia. After measuring the body weight, peripheral blood was collected with disposable vacuum blood collection (including heparin anti-coagulant) and stored at 4°C. The colon tissue was then quickly isolated and placed on ice cake; the colonic length and weight (*n* = 10) were measured; and the colonic weight index (*n* = 10) was calculated as follows: colonic weight index = colonic weight/body weight × 100%. The separated colon was longitudinally opened, and their faeces were rinsed with cold phosphate-buffered saline (PBS).

### 2.5. Pathological Analysis

The distal colon of each mouse was fixed in 4% formaldehyde solution for one week, and the colon tissue was dehydrated, embedded in paraffin, sectioned at 4 *μ*m, and then stained with hematoxylin and eosin. Next, a histological score of the colon was evaluated as double-blind analysis by two pathologists, and the degree of inflammatory infiltration and injury of the colon were evaluated according to the criteria described by Dieleman et al. The histological grading of colitis was performed as follows [14]: (1) inflammation – 0: none, 1: slight, 2: moderate, and 3: severe; (2) extent – 0: none, 1: mucosa, 2: mucosa and submucosa, and 3: transmural; (3) regeneration – 4: no tissue repair, 3: surface epithelium not intact, 2: regeneration with crypt depletion, 1: almost complete regeneration, and 0: complete regeneration or normal tissue; (4) crypt damage – 0: none, 1:1/3 damaged basal ganglia, 2:2/3 damaged basal ganglia, 3: only surface epithelium would be intact, and 4: entire crypt and epithelium lost; and (5) percentage of involvement – 1: 1–25%, 2:26–50%, 3:51–75%, and 4:76–100%.

### 2.6. Flow Cytometry Analysis

The obtained lymphocytes (*n* = 10) were incubated with fluorescein isothiocyanate (FITC) conjugated monoclonal antibodies. Flow cytometry analysis was performed on a FACSCalibur system (BD Biosciences, Franklin Lakes, NJ, USA). A lymphocyte suspension was prepared as follows: 100 *μ*L of anti-coagulant, 100 *μ*L of RPMI 1640 medium, and 1 mL of hemolysin working solution were added to fresh blood and incubated for 15 min in dark. The mixture was centrifuged at 300*g* for 5 min, and the supernatant was removed. Then, 1 mL of stain buffer was added, and the cells were rinsed and centrifuged at 300*g* for 5 min and resuspended with 100 *μ*L staining buffer and 1 *μ*g FC blocking sealant at 4°C for 8 min. Samples were then incubated with primary antibodies for 15 min at room temperature, washed with 1 mL of stain buffer, and mixed with 100 *μ*L staining buffer. The following monoclonal antibodies (mAbs) were used: PE anti-mouse CD45RA (1:200), PE-CY7 anti-mouse CD62L (1:200), APC-H7 anti-mouse CD4 (1:500), FITC anti-mouse CD8 (1:500), and Alexa Fluor 647 anti-mouse CCR7 (1:200) and second group: PE anti-mouse GL7 (1:200) and FITC anti-mouse CXCR5 (1:200; BD Biosciences, San Jose, CA) were added and incubated for 30 mins in the dark. And then it was added 1 ml of stain buffer (BD Biosciences, 554656), centrifuged to the supernatant (500 × *g*, 5 mins, 4°C), and discarded the supernatant. The 500 *μ*l stain buffer was added to the stained cells that were detected using FACSCalibur (BD Biosciences), and the detection results were analyzed by FlowJo VX10 (Treestar, Ashland, OR).

### 2.7. Western Blot Assay

Colon tissues (*n* = 6) were quickly shredded and mixed with protease inhibitor cocktails, which were randomly selected from every group. The mixture was homogenized by ultrasound on crushed ice and centrifuged at 13000*g* at 4°C for 10 min; the supernatant was extracted; and the protein concentration was determined by BCA protein assay. Equal amounts of total protein (60 *μ*g) were separated by SDS-PAGE and transferred to polyvinylidene fluoride (PVDF) membrane using a semidry transfer slot (Bio-Rad, USA). After blocking with 5% nonfat dried milk for 1 h, the membrane was incubated with proportionally diluted antibodies at 4°C overnight. The antibodies included rabbit anti-GAPDH (1:2,000), anti-BCL-2 (1:2,000), anti-BIM-1 (1:1,000), anti-caspase-3 (1:500), anti-BAX (1:1,000), anti-PP2A (1:1,000), anti-*β*-casein (1:2,500), anti-caveolin-1 (1:1,000), anti-Pim-1 (1:1,000), anti-JAK1 (1:1,000), anti-JAK3 (1:1,000), anti-PIAS1 (1:2,000), anti-PIAS3 (1:2,000), anti-Socs-1 (1:1,000), anti-P-STAT5 (1:1,000), and anti-STAT5 (1:1,000; Abcam). Then, samples were incubated with a corresponding secondary antibody (Abcam) for 1 h. After washing three times with TBST, the blots were visualized by the Proteogel imaging system (FluorChem M, ProteinSimple, USA) and quantified with the Quantity One System (Bio-Rad).

### 2.8. Statistical Analysis

Statistical analysis was performed using GraphPad Prism 8.0 (La Jolla, CA, USA), and results are presented as mean ± average standard error (SEM). Differences between groups were examined using unpaired Student's *t*-tests. *P*-values <0.05 were considered to be statistically significant.

## 3. Results

### 3.1. SSP Ameliorated DSS-Induced Colitis in Mice

**Figure 1:**
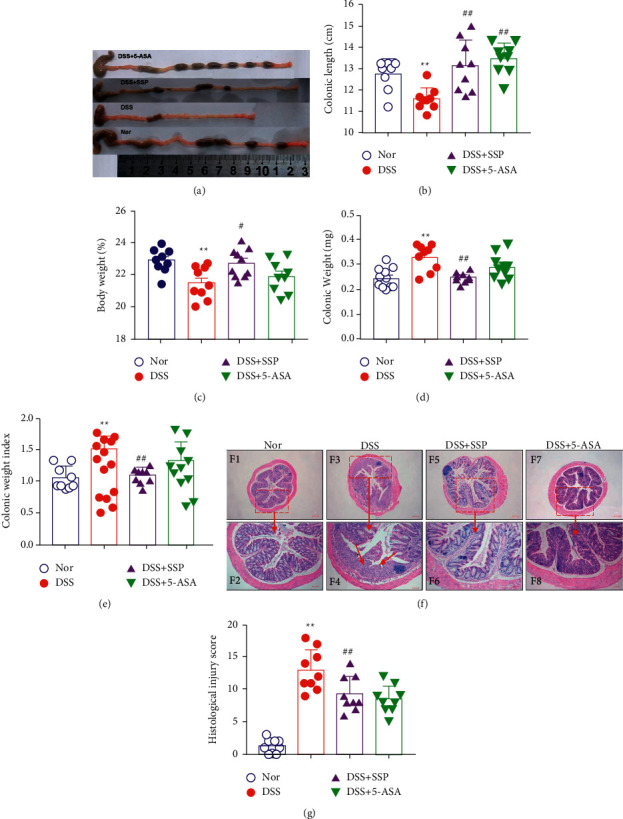
SSP ameliorates DSS-induced colitis in mice: (a) changes in colon length observed by the naked eye, (b) colonic length, (c) body weight of mice, (g) histological scores, (d) colon weight, (e) colon weight index, and (f) representational tissues were stained with hematoxylin and eosin (H&E; scale bar: 40 *μ*m and 100 *μ*m). Data are presented as mean ± SEM (*n* = 10). ^*∗*^*P* < 0.05 and ^*∗∗*^*P* < 0.01 versus nor group; ^*#*^*P* < 0.05 and ^*##*^*P* < 0.01 versus DSS group.

DSS-induced mice colitis is an alternative and classic model widely applied to study IBD treatment. In this study, mice were treated with 3% (*w*/*v*) DSS solution for 7 days to induce ulcerative colitis. As expected, 2 days after administration of 3% DSS, the weight of mice in the DSS group began to gradually decrease and mice began showing poor spirits, hunched posture, reduced mobility, and reduced diet. From the 5^th^ to 7^th^ day, the above-reported signs became more obvious, and loose and bloody stools were observed. On the 8^th^ day, mice were treated with SSP and 5-ASA for 7 consecutive days. After treatment, shortened colon length was rapidly restored in DSS + SSP and DSS+5-ASA groups compared with the DSS group (Figures 1(a) and 1(b); *P* < 0.01). At the same time, the colon weight (Figure 1(d)) and colon weight index (Figure 1(e)) were significantly reduced (*P* < 0.01). The body weight of mice also showed a recovery trend in the DSS + SSP group (*P* < 0.01); it was higher in the DSS + 5-ASA group compared to the DSS group (Figure 1(c)) in the last day (*P* < 0.01).

Microscopic evaluation showed that DSS-induced colitis was characterized by decreased glands, loss of mucus layer, and extensive inflammatory cell infiltration into the lamina propria, especially neutrophils, intestinal epithelial erosion, and even ulceration. In contrast, SSP treatment obviously ameliorated the pathological symptoms (Figure 1(f)). Moreover, the histological scores in the colon of mice from the DSS group were significantly higher than that in the Nor group, and the histological scores in DSS + SSP and DSS + 5-ASA groups were significantly lower than that in the DSS group (Figure 1(g); *P* < 0.01). These results demonstrated that SSP has a therapeutic role for DSS-induced colitis in mice.

### 3.2. SSP Promotes CD4^+^ and CD8^+^ Expressions on TSM Cells in Colitis Mice

**Figure 2:**
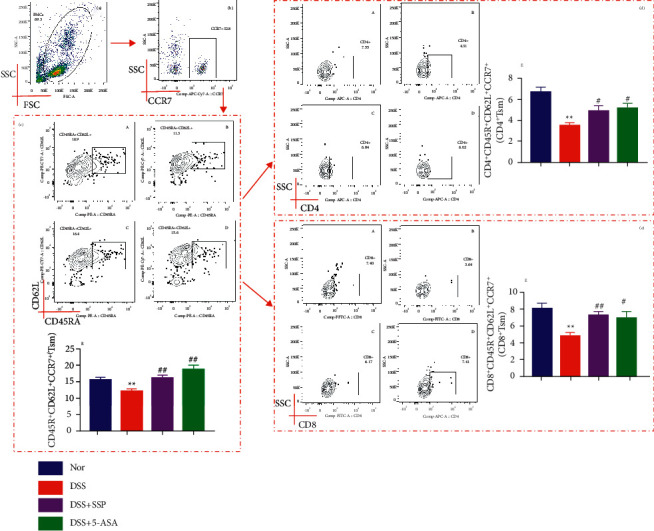
SSP promotes CD4^+^ and CD8^+^ expressions on TSM cells in colitis mice. Peripheral blood cells were isolated from mice and stained with different fluorescent antibodies; the cells were gated sequentially on living lymphocytes and CCR7^+^ and then on CD62L^+^ and CD45RA^+^ cells. The proportion of CD45RA^+^CD62L^+^CCR7^+^ TSM cells was determined. Then, Tcm cells were gated on CD4 and CD8, and the proportion of CD4^+^ TSM cells and CD8^+^ TSM cells in lymphocytes was analyzed. (a) Total lymphocytes in peripheral blood. (b) Relative percentages of CCR7^+^ labeled lymphocytes. (c) Relative percentages of CD45RA^+^CD62L^+^CCR7^+^ T cells in UC mice pre- and post-treatment. A: nor group, B: DSS, C: DSS + SSP, D: DSS + 5-ASA, and E: CD45RA^+^CD62L^+^CCR7^+^ T cells level. (d) Relative percentages of CD4^+^ TSM cells in UC mice pre- and post-treatment. A: nor group, B: DSS, C: DSS + SSP, D: DSS + 5-ASA, and E: CD4^+^CD45RA^+^CD62L^+^CCR7^+^ T cells level. (e) Relative percentages of CD8^+^ TSM cells in UC mice pre- and post-treatment. A: nor group, B: DSS, C: DSS + SSP, D: DSS + 5-ASA, and E: CD8^+^CD45RA^+^CD62L^+^CCR7^+^ TSM cells level. Data are presented as mean ± SEM (*n* = 10). ^*∗*^*P* < 0.05 and ^*∗∗*^*P* < 0.01 versus nor group. ^*#*^*P* < 0.05 and ^*##*^*P* < 0.01 versus DSS group.

Memory T cells have considerable functional and phenotypic heterogeneity. The expression of chemokine CCR7 and homing receptor CD62L is the most commonly used surface marker to distinguish memory T cells and their subsets. In this study, we used CD62L and CCR7 to characterize the activation states of the cells and reflect the level of TSM. The percentage of CD45RA^+^CD62L^+^CCR7^+^ TSM cells (Figures 2(a)–2(c)) was significantly reduced in the DSS group compared to the Nor group and SSP and/or 5-ASA group (*P* < 0.01 or *P* < 0.05). Furthermore, CD4^+^ (Figures 2(a), 2(b), and 2(d)) and CD8^+^ (Figures 2(a), 2(b), and 2(e)) expressions on TSM cells were decreased in the DSS group compared with the these in the Nor group, DSS + SSP group, and DSS + 5-ASA group (*P* < 0.01 or *P* < 0.05). These results indicate that SSP promotes the expressions of CD4^+^ and CD8^+^ on the surface of CD45RA^+^CD62L^+^CCR7^+^ TSM cells in colitis mice.

### 3.3. SSP Regulates the Subpopulations of CD4^+^ TSM Cells in Colitis Mice

**Figure 3:**
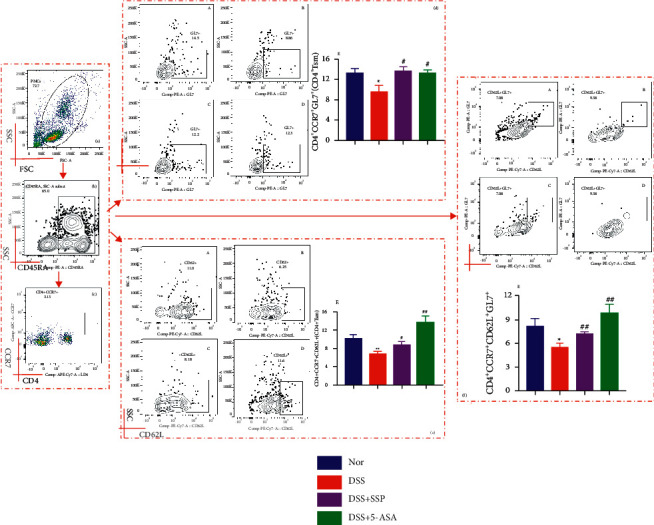
SSP regulates CD4^+^ TSM cells and their subsets in colitis mice. After being stained with different fluorescent antibodies, cells were sequentially gated on living lymphocytes, CD4^+^, CCR7^+^, GL7^+^, and CD62L^+^. CD4^+^CCR7^+^GL7^+^, CD4^+^CCR7^+^CD62L^+^, and CD4^+^CCR7^+^CD62L^+^GL7^+^ TSM cells were analyzed by flow cytometry. (a) Total lymphocytes in peripheral blood. (b) CD45RA^+^cells. (c) The proportion of CCR7^+^ labeled CD4^+^ TSM cells. (d) Relative percentages of CD4^+^CCR7^+^GL7^+^ cells in UC mice pre- and post-treatment. A: norl group, B: DSS, C: DSS + SSP, D: DSS + 5-ASA, and E: CD4^+^CCR7^+^GL7^+^ cells level. (e) Relative percentages of CD4^+^CCR7^+^CD62L^+^ cells in UC mice pre- and post-treatment. A: nor group, B: DSS, C: DSS + SSP, D: DSS + 5-ASA, and E: CD4^+^CCR7^+^CD62L^+^ cells level. (f) Relative percentages of CD4^+^CCR7^+^CD62L^+^GL7^+^ cells in UC mice pre- and post-treatment. A: nor group, B: DSS, C: DSS + SSP, D: DSS + 5-ASA, and E: CD4^+^CCR7^+^CD62L^+^GL7^+^ cells level. Data are presented as mean ± SEM (*n* = 10). ^*∗*^*P* < 0.05 and ^*∗∗*^*P* < 0.01 versus nor group and ^*#*^*P* < 0.05 and ^*##*^*P* < 0.01 versus DSS group.

Next, we analyzed the numbers of different subpopulations of CD4^+^ TSM cells in peripheral blood of mice by flow cytometry. In the present study, the results showed that the proportions of CD4^+^CCR7^+^GL7^+^ (Figures 3(a)–3(d)) and CD4^+^CCR7^+^CD62L^+^ (Figures 3(a)–3(c), and 3(e)) T cells in the DSS group were significantly downregulated when they were compared with the Nor group, DSS + SSP group, and DSS + 5-ASA group (*P* < 0.01 or *P* < 0.05). In addition, the proportion of CD4^+^CCR7^+^CD62L^+^GL7^+^ T cells (Figures 3(a)–3(c), and 3(f)) in colitis mice after SSP and 5-ASA treatment was significantly higher than that in those colitis mice before treatment (*P* < 0.01 or *P* < 0.05). The results suggested that SSP increased the number of CD4^+^ TSM cells and their subsets in DSS-induced colitis.

### 3.4. SSP Regulates CD4^+^ mTfh Cells and Their Subpopulations in Colitis Mice

**Figure 4:**
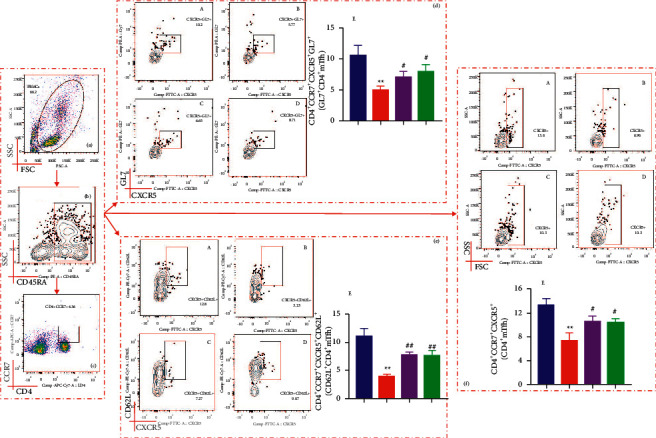
SSP regulates CD4^+^ mTfh cells and their subsets in colitis mice. After being stained with different fluorescent antibodies, the cells were sequentially gated on living lymphocytes, CD4^+^, CCR7^+^, and CXCR5^+^. The proportion of CD4^+^CCR7^+^CXCR5^+^mTfh cells was determined. Subsequently, CD4^+^mTfh cells were gated on GL7 and CD62L, and the proportion of GL7^+^CD4^+^mTfh cells and CD62^+^CD4^+^mTfh cells in lymphocytes was analyzed. (a) Total lymphocytes in peripheral blood. (b) CD45RA^+^ cells. (c) The proportion of CCR7^+^ labeled CD4^+^ T cells. (d) Relative percentages of GL7^+^CD4^+^mTfh cells in UC mice pre- and post-treatment. A: nor group, B: DSS, C: DSS + SSP, D: DSS+5-ASA, and E: GL7^+^CD4^+^cells level. (e) Relative percentages of CD62^+^CD4^+^mTfh cells in UC. A: nor group, B: DSS, C: DSS + SSP, D: DSS + 5-ASA, and E: CD62^+^CD4^+^cells level. (f) Relative percentages of total CD4^+^mTfh cells in UC. A: nor group, B: DSS, C: DSS + SSP, D: DSS + 5-ASA, and E: CD4^+^CCR7^+^CXCR5^+^cells level. Data are presented as mean ± SEM (*n* = 10). ^*∗*^*P* < 0.05 and ^*∗∗*^*P* < 0.01 versus nor group and ^*#*^*P* < 0.05 and ^*##*^*P* < 0.01 versus DSS group.

Tfh cells exist in lymphoid organs, such as germinal centers, and memory Tfh cells (mTfh) can be found in peripheral blood or local tissues. mTfh cells are also an important part of T cell memory in both mice and humans [4]. In this study, we evaluated whether cell percentages of CD4^+^CD45RA^+^CCR7^+^CXCR5^+^ (CD4^+^ mTfh) cells changed in experimental colitis. As shown in Figure 4, the percentages of CD4^+^ mTfh cells in untreated mice with colitis were reduced when they were compared to those in the Nor group, DSS + SSP group, and DSS + 5-ASA group (Figures 4(a)–4(c), and 4(f); *P* < 0.01 or *P* < 0.05). Furthermore, the change in the numbers of different subpopulations of CD4^+^ mTfh cells in colitis mice is associated with SSP treatment. As shown in Figure 4, the levels of CD4^+^CD45RA^+^CCR7^+^CXCR5^+^GL7^+^ T cells (Figures 4(a)–4(d)) and CD4^+^CD45RA^+^CCR7^+^CXCR5^+^CD62L^+^ T cells (Figures 4(a)–4(c), and 4(e)) from the DSS group were significantly lower than those in the Nor group, but they were significantly higher than the DSS + SSP and DSS + 5-ASA groups (*P* < 0.01 or *P* < 0.05). These data indicated that SSP administration regulates the number of CD4^+^ mTfh cells and their subpopulations in DSS-induced colitis.

### 3.5. SSP Modulates Protein Levels of JAK/STAT5 Signal Molecule in Colonic Tissues

**Figure 5:**
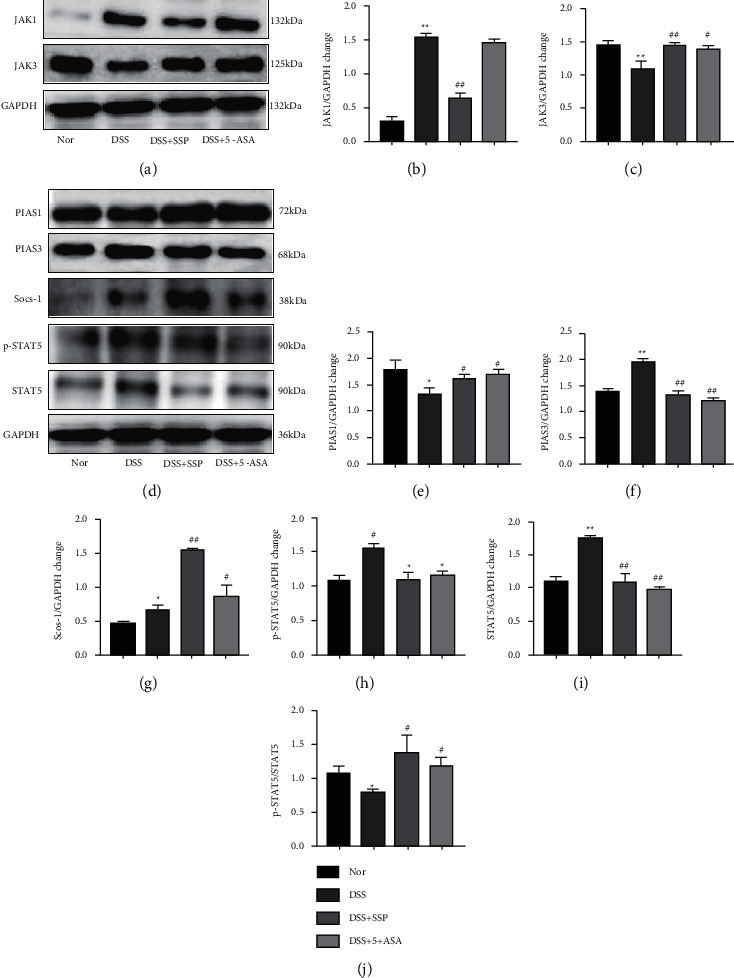
SSP modulates protein levels of JAK/STAT5 signal molecules in colonic tissues. Colon tissue homogenates were collected after SSP treatment, after which the expression levels of JAK/STAT5 signal molecule were analyzed by Western blot analysis. (a) Expression of JAK1 and JAK3; (b) quantitative analysis of JAK1; (c) quantitative analysis of JAK3; (d) expression of PIAS1, PIAS3, Socs-1, P-STAT5, and STAT5; (e) quantitative analysis of PIAS1; (f) quantitative analysis of PIAS3; (g) quantitative analysis of Socs-1; (h) quantitative analysis of P-STAT5; (i) quantitative analysis of STAT5; and (j) quantitative analysis of p-STAT5/STAT5. Data are presented as mean ± SEM (*n* = 6). ^*∗*^*P* < 0.05 and ^*∗∗*^*P* < 0.01 versus nor group and ^*#*^*P* < 0.05 and ^*##*^*P* < 0.01 versus DSS group.

The activation of members of the JAK/STAT5 signal transduction pathway is considered to be the necessary pathway for intestinal inflammation and T cell differentiation. To further explore molecular mechanisms, the related protein expression levels of the JAK/STAT5 signal were determined using Western blot. The present results showed that JAK1 (Figures 5(a) and 5(b)), p-STAT5 (Figures 5(d) and 5(h)), and STAT5 (Figures 5(d) and 5(i)) were upregulated (*P* < 0.01 or *P* < 0.05), and JAK3 (Figures 5(a) and 5(c)) was downregulated in the DSS group when compared with them in the Nor group, SS + SSP group, and DSS + 5-ASA group (*P* < 0.01 or *P* < 0.05), thus suggesting that the JAK/STAT5 signaling pathway was activated, and SSP deactivated JAK/STAT5 signal. In addition, the ratio of p-STAT5/STAT5 (Figures 5(j)) was increased after mice with experimental colitis were treated after SSP (*P* < 0.05). The results indicated that SSP could restrain the activation of the JAK/STAT5 pathway in experimental colitis induced by DSS.

Furthermore, we found that regulators of the JAK/STAT5 pathway, including PIAS1 (Figures 5(d) and 5(e)) and Socs-1 (Figures 5(d) and 5(g)) were decreased (*P* < 0.01 or *P* < 0.05), and the expression of PIAS3 (Figures 5(d) and 5(f)) was increased in the DSS group when they were compared with these in the Nor group, DSS + SSP group, and DSS + 5-ASA group (*P* < 0.01).

### 3.6. SSP Regulates the Expression of Apoptotic Genes in the Mitochondrial Pathway of Colonic Tissues

**Figure 6:**
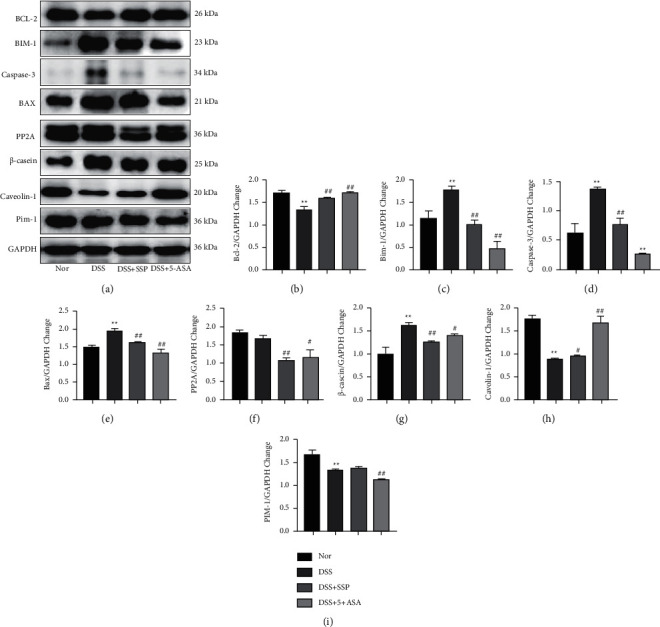
SSP modulates the other proteins in JAK/STAT5 signal in colonic tissues. The downstream proteins in JAK/STAT5 signal mainly included BCL-2, Bim-1, caspase-3, Bax, PP2A, cascin, caveolin-1, and PIM-1. (a) Expression of BCL-2, Bim-1, caspase-3, Bax, PP2A, *β*-cascin, caveolin-1, and PIM-1 in colonic tissue; (b) wuantitative analysis of BCL-2; (c) quantitative analysis of Bim-1; (d) quantitative analysis of caspase-3; (e) quantitative analysis of Bax; (f) quantitative analysis of PP2A; (g) quantitative analysis of *β*-cascin; (h) quantitative analysis of caveolin-1; and (i) quantitative analysis of PIM-1. Data are presented as mean ± SEM (*n* = 6). ^*∗*^*P* < 0.05 and ^*∗∗*^*P* < 0.01 versus nor group and ^*#*^*P* < 0.05 and ^*##*^*P* < 0.01 versus DSS group.

JAK/STAT5 signaling pathway has a significant role in the process of the differentiation and activation of memory T cells, which included its downstream proteins as BIM-1, BCL-2, caspase-3, BAX, PP2A, and so on. As shown in Figure 6, the expressions of BCL-2 (Figures 6(a) and 6(b)) and caveolin-1 (Figures 6(a) and 6(h)) were lower (*P* < 0.01 or *P* < 0.05), while the expression of BIM-1 (Figures 6(a) and 6(c)), caspase-3 (Figures 6(a) and 6(d)), BAX (Figures 6(a) and 6(e)), and *β*-casein (Figures 6(a) and 6(g)) were higher in the DSS group than in the Nor group, DSS + SSP group, and DSS + 5-ASA group (*P* < 0.01 or *P* < 0.05). Although it is not discrepant when the PP2A (Figures 6(a) and 6(f)) and Pim-1(Figures 6(a) and 6(i)) expressions in the DSS group were in contrast to them in the Nor group, their expressions were dramatically decreased in the DSS + SSP and DSS + 5-ASA groups when they were compared with them in the DSS group (*P* < 0.01 or *P* < 0.05). These data suggest that SSP effectively regulates the activation of the downstream proteins of the JAK/STAT5 pathway.

## 4. Discussion

Although the exact etiology and pathogenesis of UC still keep unclear, it is known that the abnormal immunological status induced by immunological memory disorder or unbalance of memory immune cells subsets is one of the main pathogenic factors leading to UC [14]. Many previous studies have indicated that abnormal immune memory is an important characteristic of inflammatory bowel disease, including UC [1]. According to the CCR7 and CD62L expression, the memory T cells are classified into central memory T cells (CD45RA^−^CD62L^+^CCR7^+^T cell, TCM), effector memory T cells (CD45RA^−^CD62L^−^CCR7^−^T cell, TEM), special memory T cells (CD45RA^+^CD62L^+^CCR7^+^T cell, TSM), memory T follicular cells, and so on [15]. In the present study, mice colitis was successfully induced by DSS administration. The mice developed diarrhea, bloody stool, ulcer formation, crypt disappear and abundant inflammatory cells infiltration, and so on, which is consistent with the animal model of ulcerative colitis previously reported [16]. DSS mice developed significant changes in memory T cells compared to normal control mice. The total of special memory T cells (as CD45RA^+^CD62L^+^CCR7^+^ and CD4^+^CCR7^+^CD62L^+^T cells) and memory T follicular helper cell (mTfh; as CD4^+^CCR7^+^CXCR5^+^T cells) were markedly decreased, while the expressions of CD4^+^, CD8^+^, GL7^+^, CD62L^+^ molecules on the surface of memory T cells and mTfh cells were distinctly reduced in the DSS mice, which suggested that low-level immune memory has an important role in DSS-induced colitis in the present study.

In the early stage, T cells are activated and differentiated into effector T cells. After binding with antigens, most of the effector T cells undergo apoptosis, while some become memory T cells. In the present study, as a special type of memory T cells, CD45RA^+^CCR7^+^CD62L^+^T cell can be divided into CD4^+^ and CD8^+^ two subtypes. Many studies have shown that the immune memory dysfunction induced by memory T cells is involved in the onset of UC and has become one of the main reasons for the prolonged and recurrent onset of UC [17, 18]. In 2006, although Japanese peers had found that FTY720 suppresses CD4^+^CD44^high^CD62L^−^ effector memory T cell to treat experimental colitis, the effect of memory T cells is rarely reported in the human UC [15]. These researches had indicated that memory T cell is a potential target to treat or prevent the occurrence of inflammatory bowel disease. As shown in Figures 2–4, the total of CD45RA^+^CCR7^+^CD62L^+^T cell, CD4^+^CD45RA^+^CCR7^+^CD62L^+^T cell, and CD8^+^CD45RA^+^CCR7^+^CD62L^+^T cell was decreased in DSS mice, while memory Tfh cells showed similar change, which suggests that the low-level immune memory function is one of the main pathogenetic characteristics of UC.

SSP is a classic and frequently used traditional Chinese medicine used to treat chronic ulcerative colitis (UC). In this study, we examined the effect of SSP on immune memory function in DSS mice. SSP effectively alleviated the pathological colonic mucosa damage induced by DSS and improved the total of the memory T cells (as CD45RA^+^CD62L^+^CCR7^+^ and CD4^+^CCR7^+^CD62L^+^T cells) and memory T follicular helper cell (mTfh; as CD4^+^CCR7^+^CXCR5^+^T cells). In addition, SPP increased the expression of CD4^+^, CD8^+^, GL7^+^, and CD62L^+^ molecules on their surface. These results revealed that SSP-treated UC is closely related to the restored memory T cells differentiation and function.

After acute infection or antigen clearance, the differentiation of memory T cells no longer relies on the classical antigen-recognition MHC-II pathway but mainly on intracellular signals (such as JAK/STAT and PI3K/Akt signaling pathways) and inflammatory mediators [6]. Previous studies have shown that the overactivation of the JAK/STAT signaling pathway is closely related to the pathogenesis of colitis, which seems to be involved in the regulation of physiological processes such as proliferation, apoptosis, and differentiation of various immune cells [19, 20]. The JAK/STAT signaling pathway has an important role in the cellular immune response [21]. When the receptor on the surface of T lymphocytes receives the stimulus signal, the receptor configuration changes, and this change will cause the activation of intracellular protein tyrosine kinase. This activated signal is then transmitted to the nucleus through STAT, causing the occurrence of the immune response [22]. STAT5 activation occurs primarily through the activation of JAK1 and JAK3. Yet the SOCS, PTPs, and PIAS are the main negative regulators of JAK-STAT signal [23]. Overexpression of SOCS1 leads to a decrease in p-JAKs and p-STATs; PIAS1 and PIAS3 can inhibit the binding of STATs to target gene promoters by coupling with STATs molecules and inhibit the transcriptional activity of STATs by modifying the phosphorylation sites of STATs [24]. In this study, the expressions of SOCS-1, PIAS3, and JAK3 in the colon tissues were significantly decreased in mice with colitis, while the expressions of PIAS1 and JAK1 were significantly increased, as well as the activities of p-STAT5 and STAT5 were also significantly increased compared with the Nor group. This indicates that the JAK/STAT5 signaling pathway is activated during UC.

Activation of the JAK/STAT5 signaling pathway led to low expression of the downstream target gene Bcl-2, which inhibited the anti-apoptotic effect, thereby promoting the apoptosis of memory T cells and aggravating intestinal inflammation [23]. Interestingly, we found that SSP treatment inhibited the JAK/STAT5 signaling pathway, which is consistent with the levels of T cells that regulate immune memory.

Survival and maintenance of memory T cells depend on the expression of Bcl-2 and Bim-1. Bcl-2 promotes the survival of T cells, and Bim-1 drives the death of activated T cells [25]. Bim-1, which is not bound to Bcl-2, can promote apoptosis by activating Bax and/or Bak. The balance between Bcl-2 and Bim-1 has a key role in maintaining the survival and homeostasis of memory T cells [25]. Bcl-2 and Bax can jointly activate proapoptotic factor caspase-3 [26]. Compared with the Nor group, the expression of Bcl-2 was decreased, while the expressions of Bim-1, Bax, and caspase-3 were significantly increased in the DSS group, appeared, and accompanied with memory T cells dysfunction. However, after experimental colitis was treated with SSP for 7 days, the protein expression of Bax and caspase-3 in colon tissue of experimental colitis mice was significantly downregulated, while the protein expression of Bcl-2 was upregulated. The SSP stimulated and restored the balance of memory T cells.

The expression of Pim-1 and PP2A decreased after SSP treatment. Pim-1 and PP2A are also closely related to cell differentiation. Pim-1 is a target gene of the STAT5 signaling pathway and a key kinase that regulates cell differentiation [23]. PP2A is involved in a variety of activities such as cell cycle, metabolism, and migration. Recent many studies have found that PP2A is widely involved in immune dysregulation diseases and is closely related to T cells and B cells [27].

We found that SSP treatment significantly reduced the expression of *β*-Casein and increased the expression of caveolin-1. *β*-Casein is a proinflammatory cytokine regulated by STAT5 that induces a T-cell-mediated inflammatory response [28]. Caveolin-1 is a multifunctional scaffold protein that acts as a platform for cell signal transduction and has an important role in the inflammatory response. Caveolin-1 inhibits cytokine signal transduction by inhibiting the kinase activity of JAK family members [28], induces T cell proliferation and secretion of cytokines, and has a protective role in ulcerative colitis [29].

Previous studies have reported that SSP and its effective components can inactivate the JAK/STAT signaling pathway in other diseases [10]. SSP inhibits the activation of p-STAT5, increases the expression of STAT5, and regulates the differentiation and function of Tfh cells in the treatment of IBD. However, as one of the priority drugs in the UC clinic, in the present study, it is very interesting that mesalazine effectively relieved DSS-induced pathological injury of colonic mucosa, which is not discrepant to SSP and is potentially related to modulating the memory T cell level and the activation of JAK/STAT signaling pathway. However, its mechanism is unclear. El-Mahdy and his workmates reported that mesalazine can enhance anti-inflammatory effects to attenuate progression of oxazolone-induced colitis by restoring IL-10 and ZO-1 levels and limiting IL-6/STAT-3 trans-signaling [30, 31]. Maybe, these supplied a possible effect to regulate memory T cells in the process of mesalazine treated mice colitis. The previous study has also been reported that the main effective components of SSP include evodiamine, rutaecarpine, schizandrin B, and psoralen. Others researchers' studies had shown that evodiamine inhibits the migration and invasion of colorectal cancer by downregulating the JAK/STAT signaling pathway [10]. As shown in the above analysis, SSP can be used to treat experimental colitis by JAK/STAT signaling pathway.

## 5. Conclusion

SSP can effectively treat colitis induced by DSS, thus potentially improving the status of immune memory via the JAK/STAT signaling pathway. However, this study has a few limitations. A direct correlation between the JAK/STAT5 signaling pathway and memory T cells has not been confirmed. In our next study, we will plan to use JAK^−/−^ or STAT5^−/−^ mice and affective components of SSP and JAK inhibitor to confirm the close relationship between memory T cells and UC. We also plan to use genomics, bioinformatics, and other technologies to define the mechanism SSP regulated memory T cell to treat UC.

## Data Availability

The data that support the findings of this study are openly available in the Research Square at https://doi.org/10.21203/rs.3.rs-685615/v1.

## Abbreviations

UC: Ulcerative colitis
mT cell: Memory T cell
SSP: Sishen Pill
JAK: Janus kinase
SATA: Signal transducer and activator of transcription
DSS: Dextran sulfate sodium
mTfh: Memory T follicular helper
PIAS: Protein inhibitors of activated STATs
BIM: Bcl-2-like protein 11
BAX: BCL-2 associated X
Caspase: Cysteine aspartyl proteases
IBD: Inflammatory bowel disease
CD: Crohn's disease
DC: Dendritic cell
HLPC: High-performance liquid chromatography
Tcm: Central memory T cell
SOCS: Suppressors of cytokine signaling
BCL: B-cell lymphoma
PTPs: Protein tyrosine phosphatases
PP2A: Protein phosphatase 2A.
